# Supplementary material for: Predicting visual function by interpreting a neuronal wiring diagram
Source: Nature. 2024 Oct 2;634(8032):113–23. doi: 10.1038/s41586-024-07953-5 (PMC11446822; doi:10.1038/s41586-024-07953-5)
Supplement: Supplementary file 6 — CRF and ERF predictions for individual TmY4 and TmY9 cells. Analogous to Supplementary Data 3, but for TmY target types. Shown are the top four monosynaptic pathways, the strongest pathway passing through each of the top ten intermediary types (ranking from Extended Data Fig. 7), and the trisynaptic pathway Tm1–TmY–Dm3–TmY (see the section entitled Prediction of spatial normalization). [file 41586_2024_7953_MOESM6_ESM.zip › DataS4/TmY9q/720575940630202106.pdf]

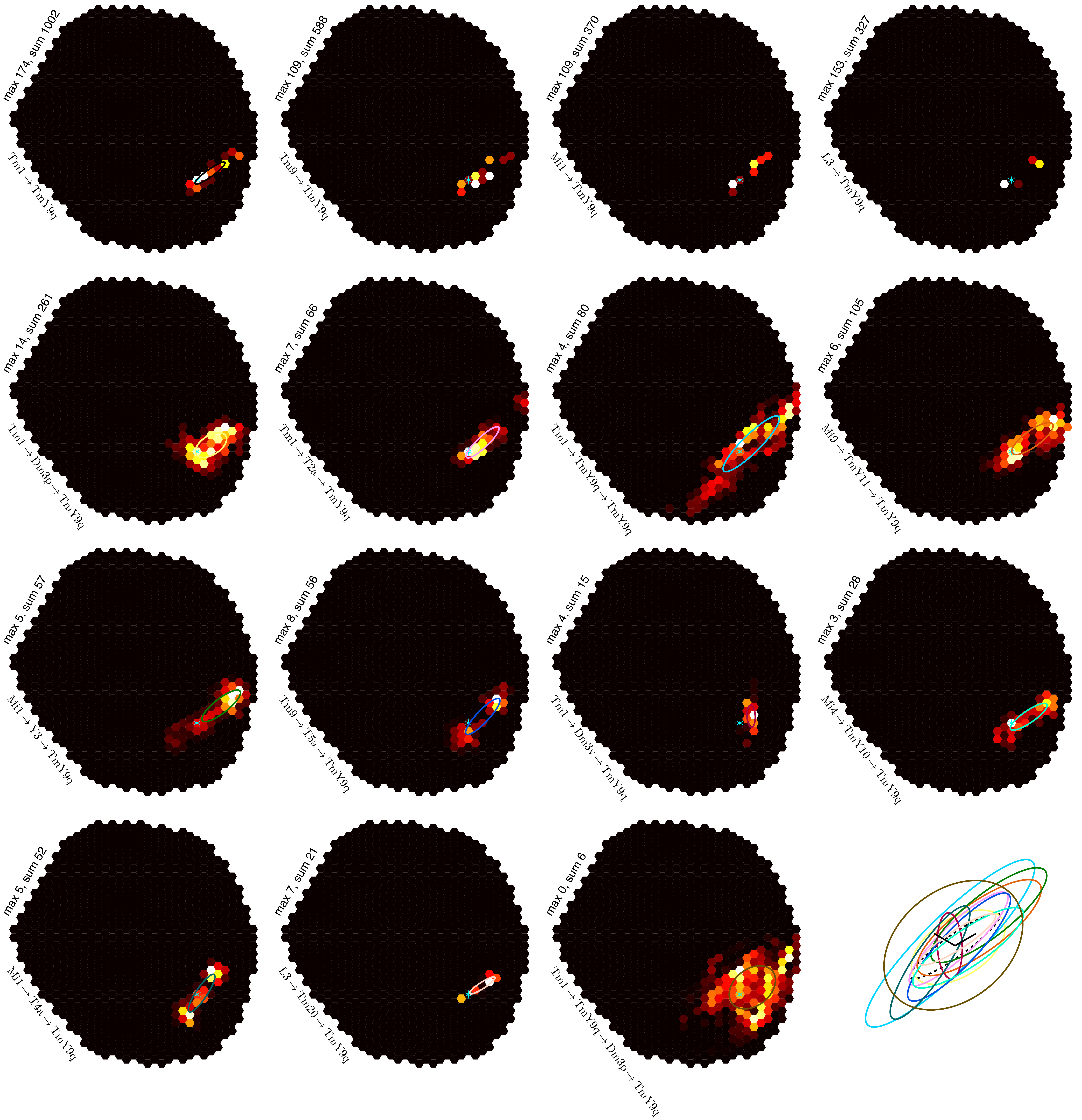

max 174, sum 1002
Tm1 → TmY9q
max 109, sum 588
Tm9 → TmY9q
max 109, sum 370
Mi1 → TmY9q
max 153, sum 327
L3 → TmY9q
max 14, sum 261
Tm1 → Dm3p → TmY9q
max 7, sum 66
Tm1 → T2a → TmY9q
max 4, sum 80
Tm1 → TmY9q → TmY9q
max 6, sum 105
Mi9 → TmY11 → TmY9q
max 5, sum 57
Mi1 → Y3 → TmY9q
max 8, sum 56
Tm9 → T5a → TmY9q
max 4, sum 15
Tm1 → Dm3v → TmY9q
max 3, sum 28
Mi4 → TmY10 → TmY9q
max 5, sum 52
Mi1 → T4a → TmY9q
max 7, sum 21
L3 → Tm20 → TmY9q
max 0, sum 6
Tm1 → TmY9q → Dm3p → TmY9q
